# Nanomechanical Mapping of Three Dimensionally Printed Poly-ε-Caprolactone Single Microfibers at the Cell Scale for Bone Tissue Engineering Applications

**DOI:** 10.3390/biomimetics8080617

**Published:** 2023-12-16

**Authors:** Marco Bontempi, Gregorio Marchiori, Mauro Petretta, Rosario Capozza, Brunella Grigolo, Gianluca Giavaresi, Alessandro Gambardella

**Affiliations:** 1 Scienze e Tecnologie Chirurgiche, IRCCS Istituto Ortopedico Rizzoli, Via di Barbiano 1/10, 40136 Bologna, Italy; marco.bontempi@ior.it (M.B.); gregorio.marchiori@ior.it (G.M.); gianluca.giavaresi@ior.it (G.G.); 2 REGENHU SA, Z.I Du Vivier 22, CH-1690 Villaz-St-Pierre, Switzerland; mauro.petretta@regenhu.com; 3 School of Engineering, Institute for Bioengineering, The University of Edinburgh, Edinburgh EH9 3DW, UK; rcapozza@exseed.ed.ac.uk; 4 Laboratorio RAMSES, IRCCS Istituto Ortopedico Rizzoli, Via di Barbiano 1/10, 40136 Bologna, Italy; brunella.grigolo@ior.it

**Keywords:** PCL, bone tissue engineering, regenerative medicine, orthopaedics, resorbable polymers, nanoindentation, scaffolds, atomic force microscopy

## Abstract

Poly-ε-caprolactone (PCL) has been widely used in additive manufacturing for the construction of scaffolds for bone tissue engineering. However, its use is limited by its lack of bioactivity and inability to induce cell adhesion, hence limiting bone tissue regeneration. Biomimicry is strongly influenced by the dynamics of cell–substrate interaction. Thus, characterizing scaffolds at the cell scale could help to better understand the relationship between surface mechanics and biological response. We conducted atomic force microscopy-based nanoindentation on 3D-printed PCL fibers of ~300 µm thickness and mapped the near-surface Young’s modulus at loading forces below 50 nN. In this non-disruptive regime, force mapping did not show clear patterns in the spatial distribution of moduli or a relationship with the topographic asperities within a given region. Remarkably, we found that the average modulus increased linearly with the logarithm of the strain rate. Finally, a dependence of the moduli on the history of nanoindentation was demonstrated on locations of repeated nanoindentations, likely due to creep phenomena capable of hindering viscoelasticity. Our findings can contribute to the rational design of scaffolds for bone regeneration that are capable of inducing cell adhesion and proliferation. The methodologies described are potentially applicable to various tissue-engineered biopolymers.

## 1. Introduction

In tissue engineering, understanding the mechanical properties of the surface of a material is crucial for guiding the design and development of natural and synthetic scaffolds [1,2,3,4]. In order to construct biopolymer-based scaffolds for bone tissue engineering, physical and chemical surface modification methods are adopted to achieve controllable modulation of the scaffold’s mechanical properties. The goal is to match tissue properties, such as in bone regeneration, enhance contact mechanics (resistance to applied loads, wear, and debris phenomena), and improve biocompatibility along with mechanical properties [5,6]. Of course, substrate stiffness cannot be regulated without changing other characteristics that are essential for biomimicry, such as surface chemistry or topography, thereby affecting surface material properties at the cell scale. Cells in contact with tissues and scaffolds sense the mechanical characteristics of their surroundings along with other physicochemical properties and convert extracellular signals into cellular responses such as differentiation and proliferation, a phenomenon called mechanotransduction. Extracellular matrix (ECM) stiffness acts as a ‘passive’ mechanical cue that counterbalances the forces exerted by cells defining their shape and anatomical localization throughout tissue homeostasis, regeneration, adaptation, and disease [3,4]. For example, the variation in stress experienced by osteoblasts on different ECM proteins in response to the same macroscopic mechanical stimuli may lead to differences in the responsiveness of osteoblasts adhered to newly resorbed bone surfaces [7,8]. These considerations emphasize the importance of measuring variations of the surface mechanical properties (i.e., typically the Young’s modulus) across lateral distances smaller or comparable to cell size (~10 µm) with sub-micrometer accuracy, hence exploring regimes of surface deformation close to real cases of cell–substrate interaction. To achieve this aim, it is necessary to decouple local (cell-scale) material properties from global (tissue-scale) properties. This requires implementing sub-micron characterization techniques along with traditional methods, such as instrumented micro- and nanoindentation, to measure the mechanical properties of tissues and substrates [3,4,9]. Atomic force microscopy (AFM)-based nanoindentation has been successfully implemented for the mechanical characterization of biopolymers and scaffolds down to sub-micrometer size. The advantage compared to traditional nanoindentation consists in its ability to measure small areas and sample surfaces at high (nanometric) lateral resolution and at loading forces in the nanonewtons range, which allows it to mechanically deform the surface of the polymer in a non-disruptive fashion. Thus, AFM has the capability to measure the near-surface mechanical properties of samples that cannot be accurately measured with traditional methods. The basic operation of AFM indentation consists of recording the relationship (force–displacement curve) between cantilever deflection and piezo movement when the nanometrically sharp tip located at its apex makes contact with the surface. In particular, under the elastic deformation regime, such a curve can be analyzed with suitable contact models to quantitatively extract the mechanical characteristics of the sample within the very first nanometers of the surface. Indeed, for shallow AFM indentations and small tip radii, the tip may predominantly sample a surface layer rather than the bulk polymer [9,10,11]. Typically, AFM provides a topographic image of the region of interest and then acquires force curves to measure the Young’s modulus (in indentation) at a given point or across the entire topography, hence generating an image of the mechanical properties of the surface with a given lateral resolution. Depending on the amount, direction, and time of applied deformation, scaffolds and tissues may exhibit complex mechanical properties at the microscale. To quantitatively assess these mechanical properties, substrates are commonly schematized as ideal (non-adhesive, elastic, linear, and isotropic) solids. However, it is well accepted that this assumption represents an oversimplification. Indeed, most natural and synthetic materials are viscoelastic, namely, the deformation depends on the rate of indentation, i.e., on the time scale of force application [3,4,10,11]. The AFM-based nanoindentation allows for the investigation of the viscoelastic response of the surface of tissues and scaffolds by regulating a set of parameters (rate of indentation, deformation, applied load) within ranges compatible with individual cell processes.

Among the most studied biopolymers for the construction of scaffolds for bone tissue regeneration, poly-ε-caprolactone (PCL) holds a prominent position. PCL is a Food and Drug Administration-approved thermoplastic polymer with excellent physicochemical properties and biocompatibility and can be resorbed by the human body with tunable degradation times. Due to its unique features, PCL has been widely used for the fabrication of scaffolds by means of different techniques [12,13]. It is also easily processable by means of advanced fabrication approaches, such as additive manufacturing techniques. Additive manufacturing techniques have indeed gathered great relevance in the tissue engineering field, since they enable the fabrication of three-dimensional scaffolds by controlled spatial deposition of materials such as natural and synthetic polymers, ceramics, or metals in a computer-aided design and modeling approach (CAD/CAM). This approach enables us to tailor the mechanical and biological properties of the fabricated structures by selecting the proper material combination and controlling internal architecture down to the microscale to match the complexity of native tissues. It also enables the inclusion of living cells in the fabrication process, in an approach defined as 3D bioprinting [14].

A wide range of additive manufacturing techniques based on different working principles, such as material jetting [15] or VAT polymerization [16], have been successfully exploited for the fabrication of engineered tissues. Nevertheless, extrusion-based techniques still represent the most common choice for thermoplastic polymers and, in particular, PCL-based formulations, due to PCL’s low melting temperature and high thermal stability. This makes it an ideal candidate for the fabrication of customized products with a multiscale-controlled internal architecture [17,18]. Despite all the advantages, the use of PCL in bone repair is still limited by its scarce bioactivity and ability to induce cell adhesion, which makes it often necessary to include specific micro- or nano-compounds to formulate composites with improved mechanical strength and capability of differentiation and osteogenesis [19,20,21].

This work exploited the potential of AFM in elucidating the surface mechanical characteristics of single fibers of 3D-printed PCL scaffolds. In particular, our study is specifically addressed to match as close as possible length scales, forces, and timescales of interaction close to individual cell–substrate interaction. It is focused on three main tasks: (i) determining experimentally the loading force corresponding to the elastic-to-plastic deformation threshold of the fiber’s surface, which is essential to performing non-disruptive nanoindentation; (ii) investigating regional variations of Young’s modulus at forces below threshold and its change with the rate of indentation (strain rate), i.e., viscoelasticity; and (iii) examining how the mechanical surface response varies with nanoindentation history, specifically when the surface is mechanically probed multiple times at the same location.

This work highlights AFM as a quantitative tool for elucidating the elastic and viscoelastic responses of PCL and other biopolymers’ scaffolds in bone tissue engineering. It establishes a methodological baseline for future investigation of scaffolds, particularly in the context of incorporating various compounds or surface treatments to enhance bioactivity and mechanical properties, especially in orthopedic applications.

## 2. Materials and Methods

### 2.1. PCL Microfibers Fabrication

**Figure 1:**
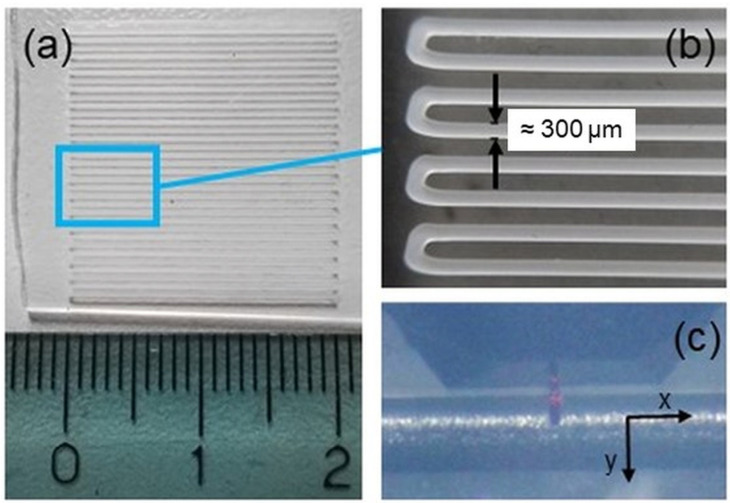
(**a**) Optical image of 3D-printed PCL microfibers onto a glass slice. (**b**) Details of the microfibers with an indication of each fiber’s thickness. (**c**) Optical image showing the orientation of the microfiber with respect to the scan directions of the AFM measurement.

PCL microfibers were fabricated by means of a hot-melt extrusion technology available in a 3D Discovery Bioprinting platform (REGENHU, Villaz-St-Pierre, Switzerland). PCL pellets of average diameter ~3 mm (MW = 80,000, Sigma Aldrich, St. Louis, MO, USA) were loaded into a stainless steel printhead. A conical, flow-optimized, stainless steel nozzle with an inner diameter of 300 μm was selected. Microfibers equivalent in total length to multiple layers of a 3D PCL scaffold were directly printed onto a glass slide. This allows the mechanical properties of the fiber to be tested at any point using AFM. The following printing parameters were used: Temperature = 110 °C; Pressure = 5 Bar; Printing Speed = 4 mm/s. To obtain a homogeneous melt, the pellets were kept at the printing temperature for 30 min before performing the process. Single-layer square structures of 20 mm × 20 mm (Figure 1a) were designed using BioCAD software (Version 1.1., REGENHU, Villaz-St-Pierre, Switzerland). Given the overall dimensions of the printed samples and the selected printing parameters, the fabrication process lasted no longer than a few minutes. For this reason, also taking into account that the process was performed at 110 °C, i.e., well within the thermal stability region of the polymer, we could confidently consider the fabricated samples as homogeneous with no significant difference in quantitative findings (see Section 2.2). An optical microscope (Eclipse 90I, Nikon, Tokyo, Japan) was used to check the correspondence between the fiber dimensions and the design parameters before further characterization. From the performed analyses, it was possible to observe that the obtained fiber diameter was (291.58 ± 2.67) μm, with less than 3% deviation from the design. The interfilament distance (center-center) was about 600 μm (Figure 1b).

### 2.2. AFM Operation

PCL microfibers deposited onto a glass slice were characterized both topographically and by the extraction of force curves by an NT-MDT (Moscow, Russia) AFM system. This setup is equipped with an upright optical microscope to properly adjust the position of the probe on the sample. The single microfiber alignment (along the x-axis) was set at our best orthogonally to the slow scan direction (i.e., along the y-axis, Figure 1c).

**Figure 2:**
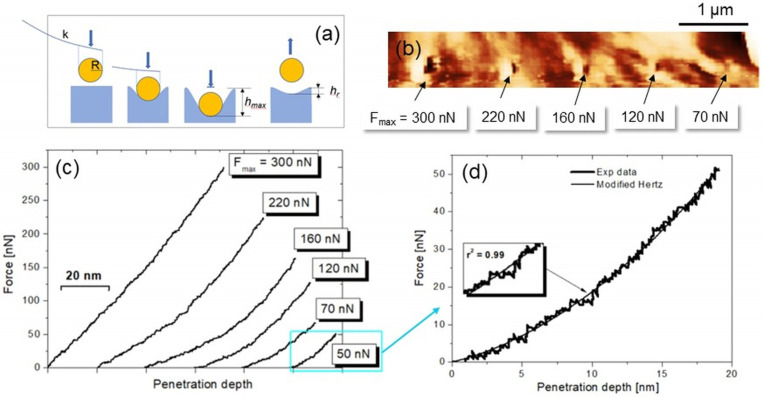
(**a**) Scheme of the indentation by a spherical indenter of radius R with indication of h_max_ and h_r_. (**b**) 5 µm × 1 µm topographic image displaying five nanoindentations points taken at F_max_ decreasing from 300 nN to 70 nN. (**c**) Force curves corresponding to indentations in (**b**); curves were shifted horizontally for better clarity. (**d**) Force curve corresponding to F_max_~ 50 nN and its corresponding fitting.

**Figure 3:**
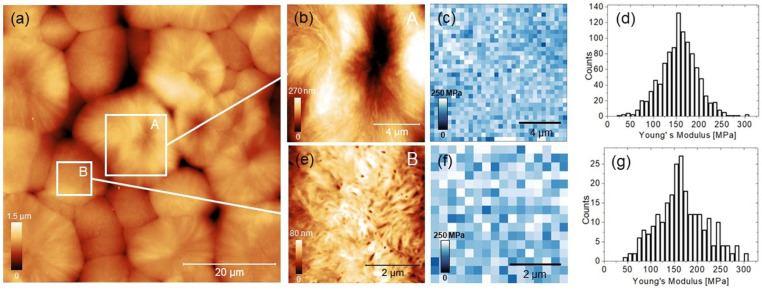
(**a**) A 60 µm × 60 µm topographic image with evidenced A- and B-type regions. (**b**) A 10 µm × 10 µm zoomed-in image of the A-type region. (**c**) Young’s modulus map (32 × 32 pixels) acquired within the topography in (**b**). (**d**) Statistical distribution of the values extracted from the map in (**c**). (**e**) A 5 µm × 5 µm zoom-in image of the B-type region. (**f**) Young’s modulus map (16 × 16 pixels) acquired within the topography in (**e**). (**g**) Statistical distribution of the values extracted from the map in (**f**).

NSG10 cantilevers (NT-MDT, Moscow, Russia) with resonant frequency in the range 140–390 kHz were used for both topographies (resolution 512 × 512 pixels in tapping mode of operation) and nanoindentations. Two cantilevers with stiffness *k* = 9.18 N/m and 10.2 N/m were used. *k* was measured according to a procedure implemented into the acquisition software (NOVA, MT-MDT, Moscow, Russia) [22]. In nanoindentation mode, the measure of the cantilevers’ deflection sensitivity was used to convert force–displacement curves into force–penetration depth curves, which are the ones used for elaboration. Such conversion was carried out from a set of indentation curves previously obtained on a clean and nanometrically flat silica slice (~80 GPa in stiffness) using cantilevers with stiffness similar to the present work; such procedure allows non-invasive calibration of a cantilever, i.e., without causing tips damages. Before and after mapping, tip integrity was checked via z-axis calibration on a TGS1 calibration grating (NT-MDT, Moscow, Russia; grid TGZ1 with height (21 ± 1) nm). According to the manufacturer, the tip apex is spherical with a curvature radius of R = (10 ± 2) nm. AFM measurements taken at different locations along the fiber, as shown in Figure 1a,b, displayed comparable morphological features. Correspondingly, moduli measured at an indentation rate of 200 nm/s (i.e., the same used in Figure 2 and Figure 3) were consistent within a 10% deviation.

Several survey curves were taken at random positions on the surface before starting map acquisition to verify that the noise-to-signal ratio was acceptable. Several curves acquired at equally spaced points along the x- and y-axes within a given square or rectangular topographic image constitute a map of force–penetration depth curves (force mapping of the sample) [23,24]. The spatial resolution of the map is given by the distance between the single indents; the spatial resolution of the maps shown here is 250–300 nm.

### 2.3. Extraction of Young’s Modulus from Experimental Indentation Curves

The Young’s modulus was calculated from each force–penetration depth curve by a modified Hertzian-like contact model that accounts for elastic deformation of the surface, while ideal conditions (negligible adhesion forces, isotropy, and homogeneity) at each point of indentation are assumed [25,26]. Given that we are interested in testing the very first nanometers of surface, such a model was chosen in that it was specifically designed for a spherical indenter and penetration depths similar to or bigger than the radius of the indenter. A more detailed description of the model used here and of the procedure of extraction of the moduli is reported in previous studies of this group [22,27]. It is straightforward that depths of 10s nm are much smaller than the fiber thickness; thus, the measured Young’s modulus is not significantly affected by bulky contributions coming from a single fiber or, eventually, from overlapping multiple fibers in a 3D fashion.

## 3. Results

### 3.1. Determination of the Elastic-to-Plastic Deformation Threshold

Measuring the viscoelastic properties of biopolymers by AFM-based nanoindentation is challenging, as the application of existing viscoelastic contact models to experimentally obtained deformation curves can be difficult due to material-specific characteristics. In practice, the intrinsic structural complexity of the polymer may induce non-elastic deformation and long-term relaxation processes of the surface [28,29]. A viable approach for measuring viscoelasticity is to monitor the Young’s modulus at different rates of indentation (strain rate), assuming ideal conditions at each point of indentation [28]. This implies a preliminary assessment of the loading force capable of achieving non-disruptive indentation, which is required for the correct use of elastic contact models used for data elaboration and the extraction of numerical results [28]. In Figure 2a, the nanoindentation operation by a sphere of radius R is sketched. The maximum penetration depth reached (h_max_) corresponds to the maximum loading force applied (F_max_). After the indentation, a residual depth h_r_ may be observed as consequence of non-elastic deformation of surface. F_max_ could be regulated via software before each indentation, and the corresponding h_max_ was then extracted from each curve. Figure 2b displays a topographic image acquired after five single nanoindentations carried out along a line at F_max_ decreasing from 300 nN (first on the left) to 70 nN (last on the right). The distance between the indents was about 1 µm, while the indentation rate was set at 200 nm/s. The five indentations and the image acquisition took ~5 min. The force vs. indentation depth curves corresponding to the five nanoindentations are reported in Figure 2c. A residual indentation depth h_r_ is still visible in the topography at F_max_ = 70 nN, while forces around 50 nN did not cause any measurable topographic modification. Thus, such force represents the elastic-to-plastic threshold of the polymer in our experiments. A representative curve taken at ~50 nN, reported in Figure 2c, is then highlighted (Figure 2d). It corresponds to a maximum penetration depth of h_max_~18 nm, which is comparable to the indenter’s size. This condition fulfills the geometrical constraint of the model used here to fit the force curves, which indeed agrees very well with the experimental one (r^2^ ≥ 0.99). Data related to the indentations of Figure 2 are provided in Table 1, where the various F_max_, h_max_ and h_r_—the latter measured from the topography acquired after the indentations—are collected.

The discrepancies between h_max_ and h_r_ suggest that partial recovery of the deformation took place, according to previous observations [22,28,29]. However, the violation of the thumb’s rule “the greater the load, the greater the depth” observed at 120 nN and 160 nN suggests that the recovery mechanism is not straightforward, i.e., as anticipated in the Introduction section, an inhomogeneous mechanical response of the surface may occur within this force regime. In the following, we first characterize regional variations in the Young’s modulus below the 50 nN threshold. Then, we vary the indentation rate to assess the viscoelastic properties of the surface. It is important to note that the above technique does not account for the limit of covalent bond scission (around several nN in polymers), which may cause minor damage at the nanoscale [30].

### 3.2. Mapping the Young’s Modulus

A representative 60 µm × 60 µm topography of PCL is reported in Figure 3a. Here a twofold spatial distribution of the polymeric chains was evidenced: A-type regions, or “roses”, from 10 to 30 µm large, previously observed by other authors [31], and B-type regions, typically smaller than 12 µm; these latter are clearly distinguishable, for example, as four flattened lobes at the bottom of the image. A zoomed-in 10 µm × 10 µm image of A is shown, along with the corresponding 32 × 32 Young’s modulus map acquired within the topography (Figure 3b,c). The statistical distribution of moduli extracted directly by the map is also reported (Figure 3d). At the present level of spatial resolution (~300 nm), there is no clear relationship between the features of the topographic image and the corresponding map of moduli. The B-type region is visible in detail in the 5 µm × 5 µm image of Figure 3e. Yet, there are no strong patterns in the spatial distribution of moduli (Figure 2f), and the statistical distribution of values (Figure 3g) is similar to the A region. The central tendency of both distributions is around (160 ± 5) MPa, which is compatible with probing the near-surface mechanical characteristics of the polymer and previous literature [32,33,34].

### 3.3. Viscoelasticity

**Figure 4:**
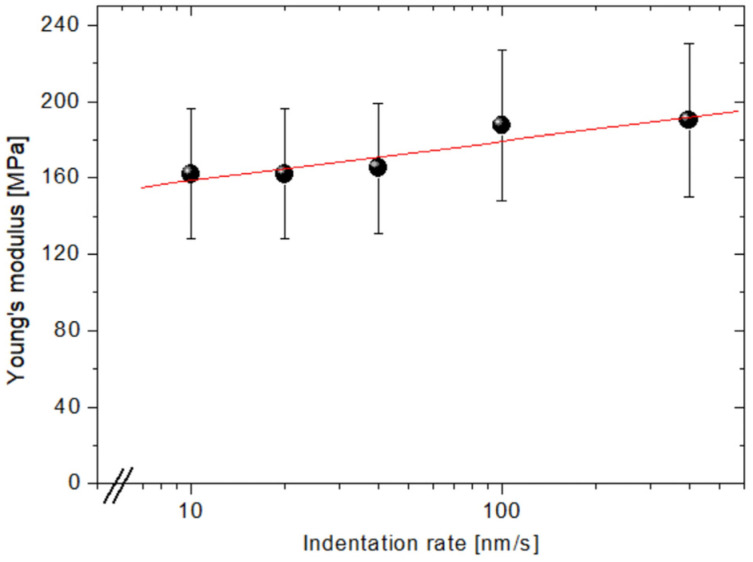
Semi-Log plot of the Young’s modulus as a function of the indentation rate and the corresponding linear fitting (red line). The indentations were carried out on non-overlapped surface points.

In practical cases, creep phenomena (i.e., the relative change in the indentation depth while the applied load is kept constant) may occur, especially at low strain rates [34]. For this reason, we first probed the local mechanical response at different (i.e., non-overlapped) surface locations. In Figure 4, each point results from averaging Young’s moduli obtained by 20 nanoindentations on as many non-overlapped points (minimum distance between points 1 µm) taken in a range of indentation rates from 10 nm/s to 400 nm/s. Significantly, we observed a linear increase in moduli (maximum 18%) with the logarithm of the indentation rate (*p* = 0.024).

Then, we conducted a separate analysis for the repeated indentation of a specific region. Specifically, we studied how the measured moduli depended on the history of nanoindentation, including the rates at which the curves were collected in either a fast-to-slow, slow-to-fast, or random manner.

### 3.4. Dependence on the History of Nanoindentation

**Figure 5:**
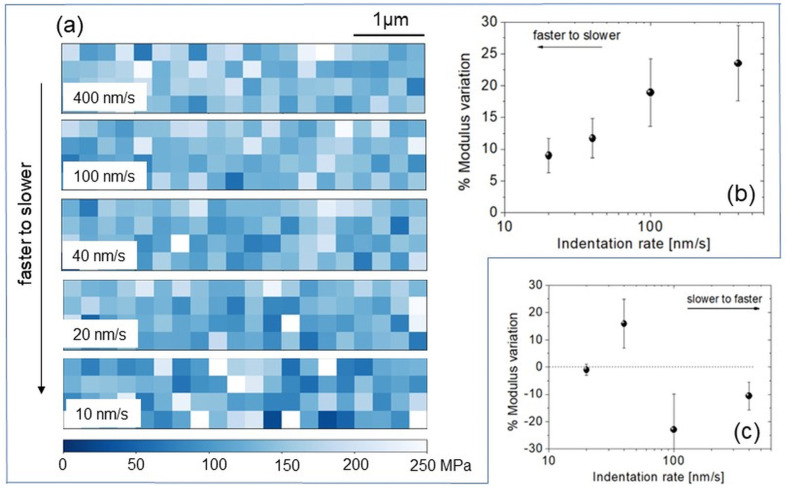
(**a**) Young’s modulus maps (5 µm × 1 µm) taken upon the same topographic region at a decreasing rate from 400 nm/s to 10 nm/s and (**b**) corresponding averaged moduli variation (%) with respect to the value acquired at the slowest rate (i.e., 10 nm/s). (**c**) Averaged moduli variation corresponding to a rate varying increasingly from 10 to 400 nm/s.

Five Young’s modulus maps (20 × 4, Figure 5a) were acquired within the same 5 µm × 1 µm-large region. The indentation rates used for each map were, respectively, 400, 100, 40, 20, and 10 nm/s (from fast to slow nanoindentations). The averaged Young’s moduli extracted from each map are reported as a function of the indentation rate (Figure 5b). Here, the value measured in correspondence of the lowest rate (10 nm/s) was taken as reference, so one can see a relative increase in moduli (up to about 30%) when the fast-to-slow rate sequence is adopted. On the other hand, when the curves were acquired according to an increasing time scale, i.e., from a slow to a fast rate, the trend of Figure 5b was not repeated (Figure 5c). In general, if a low indentation rate (10 nm/s or 20 nm/s) is applied first, any random sequence at different indentation rates will produce a scattered plot like Figure 5c. Therefore, low indentation rates produced an apparently nonreversible modification of the surface, or “memory effect”, which can hinder viscoelasticity.

## 4. Discussion

### 4.1. Significance of the Study

Several studies have demonstrated that bulk properties alone cannot define the mechanical behavior of scaffolds comprehensively, but surfaces and interfaces also play an important role in response at the cell scale [9]. For example, in 3D-printed PCL scaffolds, it has been proven that the mechanical microenvironment of the cells is provided by the local features of the surface, while the overall mechanical properties of the scaffold have little impact on the behavior and fate of individual cells [35]. Thus, detecting local gradients in scaffold elastic and viscoelastic properties has important ramifications for understanding the behavior of cells as a function of their extracellular mechanical environment. In this respect, the major advancement proposed here relies on the use of AFM as a microscale mechanical sensing technique to test PCL at indentation parameters relevant for cell interactions, namely (i) lateral micrometric and vertical nanometric scales comparable to cell dimensions and components [36]; (ii) nanonewtons-sized indentation forces comparable to the forces exerted by cells [3,4], e.g., by fibroblasts [37]; and (iii) indentation rates compatible with the individual cell-ECM dynamics [3,4]. Here, investigation at the nano-/microscale has focused on the determination of the Young’s modulus at the very first nanometers of surface, hence on the measure of the mechanical response of the surface rather than the bulk [25,30]. This may naturally lead to discrepancies with quantitative results extracted from traditional literature on mechanical tests on synthetic substrates [3]. To the best of our knowledge, there are no studies that relate the near-surface mechanics of scaffolds with (bone) cell fate. In this light, our study provides—besides an unprecedented characterization of PCL—a methodology that specifically addresses the mechanical environment actually sensed by cells.

### 4.2. Significance of the Results

The present study employed PCL as a bed test to explore the potential of AFM-based nanoindentation in detecting gradients of the elastic modulus at the surface. The relative absence of gradients in the spatial distribution of moduli observed in this study is not surprising, and is compatible with the paucity of mechanical stimuli capable of inducing bone tissue integration and bioactivity in pure PCL scaffolds, as demonstrated, for example, by culturing mouse calvaria-derived pre-osteoblastic cells [38].

In a recent study of this group, non-disruptive AFM nanoindentation at the micron scale was carried out on medical-grade polyethertherketone (PEEK) [22]. Similar to the present results, the distribution of the topographic asperities in PEEK seemed quite irrelevant with respect to the distribution of moduli. On the other hand, our findings suggested that in PEEK, the amorphous and crystalline phases were distinguishable at the surface as localized and spatially distinct distributions of moduli. In PCL, the absence of such localization—in the limit of spatial resolution investigated—may arise from the prevalence of the amorphous over the crystalline phase, which depends on printing parameters and processes and influences the mechanical performance of the polymer [19,39].

Recently, the incorporation of ceramics such as bioactive glasses into the PCL matrix has yielded a class of hybrid biomaterials with remarkably improved mechanical properties and enhanced bioactivity that are suitable for the effective treatment of bone injuries [19,35,40,41,42]. Moreover, the development of gradient scaffolds, which can be either isotropic or anisotropic, has drawn attention. These scaffolds aim to mimic the ECM by incorporating gradual transitions in structural, compositional, and/or mechanical characteristics, the latter being typically quantified by measuring the sample’s elastic (Young’s) modulus [8,43,44,45]. What is stated above encourages the exploration of different PCL chemical formulations, particularly when various scaffolds and substrates are tested to understand and tune mechanical, morphological, and chemical surface properties that govern cell fate [46].

Viscoelastic phenomena during macroscale nanoindentation of PCL were described by previous literature and related to a change in the internal friction component within the crystalline and amorphous phases, where the friction increases with strain rate as a consequence of the higher local deformation speed [32]. The resulting increase in stiffness matches well with our results in Figure 4. On the other hand, Tranchida et al. assessed the near-surface viscoelasticity of poly(propylene glycole)s at time scales and Young’s moduli compatible with our data, obtaining similar results [28].

Furthermore, Tweedie et al. suggested that in amorphous polymers, nanoindentation can induce stiffening of the surface layer in contact with the tip at depths lower than 50 nm [47]. Such stiffening is ultimately related to the creation of a mechanically unique interfacial region between the probe and the polymer, but it did not hinder the expected viscoelastic behavior when the fast-to-slow sequence rate was applied (Figure 5b). On the other hand, using low indentation rates may increase the residual imprint [28], causing h_r_ ~ h_max_. Repeated nanoindentations onto or in the vicinity of such irreversibly modified surface locations introduce a dependency on the nanoindentation history that may hinder the viscoelasticity (Figure 5c).

Nevertheless, we deem that various factors, such as irreversible modifications at the nanoscale and/or cross-linking phenomena between different points of nanoindentation, may concur to originate the effects observed, whose deeper analysis is beyond the scope of this study. Further drawbacks of the AFM measurement, such as inhomogeneities in the tip-sample contact and modifications of the tip shape during scanning, may affect the numerical accuracy and reproducibility of the results. Nevertheless, accurate characterization of the tip geometry is generally very difficult to accomplish, except in cases where its dimensions exceed hundreds of nanometers [20,21,23]. A suitable experimental setting (different cantilever stiffness, tip shape, etc.) may allow the methodology developed here to be adopted to extract the mechanical properties of PCL scaffolds with different formulations or even scaffolds based on different biopolymers. In these cases, it is underlined that different experimental contexts will require models ad hoc to fit the force–indentation depth curves, whose strict compliance should always be verified, as has been carried out in this study (Figure 2c).

For shallow nanoindentations, changes in the surface chemistry and structure of scaffolds resulting from compositional changes and/or the use of different printing techniques would be expected to have a greater influence on the gradients of elastic and viscoelastic characteristics than purely geometric changes such as the exploitation of different fiber diameters [9]. Thus, further investigations will involve the use of micrometer-resolved Raman spectroscopy to elucidate the relationship between mechanical properties and the surface composition of polymer scaffolds.

## 5. Conclusions

Since a complex mechanical interaction between cells and ECM exists on a microscopic scale, accurate mapping of the substrate’s mechanical characteristics is essential to the rational design of functional scaffolds capable of promoting bone cell regeneration and proliferation via controllable modulation of their Young’s modulus. This work promoted AFM-based nanoindentation as a valuable means to mimic the dynamic of individual cell–substrate interaction in terms of applied forces and timescales. The application of an AFM-based nanoindentation method specifically addressed to probe the mechanical properties of 3D-printed PCL microfibers was able to reveal (i) spatial variations of Young’s modulus with submicrometric accuracy and (ii) viscoelasticity and nanoindentation history dependence. The results obtained here encourage the application of the method to other polymeric systems and scaffolds for bone tissue engineering.

## Figures and Tables

**Table 1:** Relevant parameters (F_max_, h_max_, and h_r_) corresponding to the indentations and related curves in Figure 2b,c.

Maximum Force F_max_ (Curve) [nN]	Maximum Penetration Depth h_max_ (Curve) [nm]	Residual Depth After 5 min h_r_ (Image) [nm]
300	72	67 ± 11
220	66	15.5 ± 5.5
160	61	7.5 ± 2.5
120	45	12.5 ± 5.5
70	29	3.5 ± 2.5
50	21	0 ± 2

## Data Availability

The data presented in this study are available on request from the corresponding author.
